# Epidemiology of malaria and district-level factors associated with malaria elimination in Sumatra region, Indonesia: a retrospective analysis of surveillance data

**DOI:** 10.1186/s12916-026-05104-3

**Published:** 2026-07-29

**Authors:** Karina D Lestari, Henry Surendra, Bimandra A Djaafara, Ihsan Fadilah, Lenny L Ekawati, Hermawan Susanto, Hellen D Prameswari, Sri B Fajariyani, Dedy Supriyanto, Herdiana Herdiana, Eamon Conway, Pimphen Charoen, Saranath Lawpoolsri, Wirichada Pan-ngum, Iqbal R F Elyazar

**Affiliations:** 1 Oxford University Clinical Research Unit Indonesia, Faculty of Medicine, Universitas Indonesia, Jakarta, Indonesia; 2 Department of Tropical Hygiene, Faculty of Tropical Medicine, Mahidol University, Bangkok, Thailand; 3 Infectious and Tropical Diseases Epidemiology, Public Health Program, Monash University Indonesia, Tangerang, Indonesia; 4 Saw Swee Hock School of Public Health, National University of Singapore, Singapore, Singapore; 5 Centre for Tropical Medicine and Global Health, Nuffield Department of Medicine, University of Oxford, Oxford, UK; 6 Malaria Working Group, Ministry of Health, Jakarta, Indonesia; 7 World Health Organisation, Country Office for Indonesia, Jakarta, Indonesia; 8 Centre for Online Health, Medical School, Faculty of Health, Medicine and Behavioural Sciences, The University of Queensland, Brisbane, QLD Australia; 9 Walter and Eliza Hall Institute of Medical Research, Melbourne, Australia; 10 Mahidol Oxford Tropical Medicine Research Unit (MORU), Bangkok, Thailand

**Keywords:** Epidemiology, Malaria, Sumatra, Elimination, Spatial analysis

## Abstract

**Background:**

As transmission declines, malaria becomes more spatially and demographically concentrated. This study investigated individual-level malaria characteristics and district-level factors associated with elimination in all 154 districts in Sumatra, Indonesia.

**Methods:**

We analysed individual-level data of 13,386 reported malaria cases from 2019 to 2021. *P. falciparum* and *P. vivax* were confirmed by microscopy or rapid diagnostic test, while probable *P. knowlesi* was defined as microscopically identified *P. knowlesi*. Cases were classified as imported if they reported travel to endemic region within 2–4 weeks. Multivariable logistic regression with province as a random effect was used to examine individual-level factors associated with imported cases, and district-level socioeconomic and environmental factors associated with elimination. Spatial clustering of indigenous cases was assessed using Global Moran’s *I* and SaTScan.

**Results:**

Among reported cases, 71.8% (9,617) were *P. vivax*, 18.4% (2,467) *P. falciparum*, and 2.8% (369) probable *P. knowlesi*. Of 11,390 investigated cases, 8,748 (76.8%) were indigenous, 1,741 (15.3%) imported, and 901 (7.9%) unclassified. Imported cases were associated with older age (adjusted odds ratio 1.79, 95%CI 1.42–2.25 for 15–24 years old, and 1.97, 1.59–2.46 for 25–64 years old, compared to < 15 years old), males (1.70, 1.43–2.03), mobile and migrant population-related occupations (1.46, 1.22–1.76), diagnosed at hospitals or clinics (2.32, 1.85–2.91), infected with *P. falciparum* (1.48, 1.22–1.80), and hospitalised (15.29, 12.66–18.46). At the district level, a higher human development index (1.15, 1.01–1.34), lower historical annual parasite incidence (0.46, 0.29–0.69), and fewer endemic neighbours (0.78, 0.67–0.89) were associated with a higher likelihood of elimination. Spatial analysis revealed clustering tendencies, with localised clusters for *P. falciparum*, *P. vivax*, and probable *P. knowlesi*.

**Conclusions:**

Dynamics of imported malaria, heterogeneous clusters of local transmission, and lower district-level human development index challenged elimination efforts. This highlights the need for strengthened surveillance and targeted interventions.

**Supplementary Information:**

The online version contains supplementary material available at https://doi.org/10.1186/s12916-026-05104-3.

## Background

Malaria is a vector-borne disease that remains one of the main global health issues, particularly in low- and middle-income countries. The World Health Organisation (WHO) reported that the global number of estimated malaria cases has increased from 236 million in 2019 to 263 million in 2023 [1]. Despite substantial efforts and control strategies, progress towards elimination has stalled and is currently off track. Key challenges include funding shortfalls, the spread of drug and insecticide resistance, and the impact of climate change on vector distribution and transmission dynamics [1]. Furthermore, the emergence of zoonotic *P. knowlesi* infection [2] and inequalities in healthcare access [3] further complicate malaria control and elimination efforts.

Indonesia has been one of the major malaria hotspots in the Southeast Asia region, with an estimated 1.1 million cases and 1.9 thousand deaths in 2023, according to the WHO [1]. The National Malaria Program (NMP) reported that 401 of 514 districts had been declared malaria-free in 2024, leaving about 7% of Indonesia’s population (21 million people) at low to moderate risk and 2% (4.5 million people) at high risk of malaria infections [4]. Despite this considerable reduction in malaria incidence over the past decade, Indonesia’s goal for achieving malaria elimination in 2030 still encounters challenges. The decreasing trend has slowed since 2014, and case resurgences have also been observed [5]. Eliminating malaria in Indonesia is hindered by challenges of limited health systems capacity, uneven distribution of healthcare facilities, the complexity of detecting and controlling multi-species infections, and the difficulty of preventing resurgence driven by imported malaria cases [6]. A recent review suggested that there were pockets of malaria transmission that persisted in remote areas and among mobile and migrant (MMP) populations in the western part of Indonesia [7].

As one of Indonesia’s largest islands in the western region, Sumatra faces complex challenges in eliminating malaria. For example, the emergence of *P. knowlesi* infections in humans was recently confirmed by studies conducted in Aceh and North Sumatra provinces [8, 9]. NMP has also identified difficulties in reducing malaria among Suku Anak Dalam, an indigenous community in Jambi province [6]. Moreover, malaria-free districts in Sumatra are at risk of malaria importation and reintroduction due to the high movement of the MMP, including agricultural workers, forest workers, and miners [10], who often engage in outdoor and overnight activities carried out in malaria-endemic areas. Finally, environmental factors such as altitude, distance from forest, and rainfall were associated with malaria in South Sumatra province [11].

While some districts in Sumatra have successfully interrupted transmission, others continue to sustain malaria, reflecting substantial heterogeneity. It has also been observed that as malaria transmission rates decrease, case distribution becomes more concentrated, clustering within populations that share similar social, behavioural, and geographical risk factors [12]. Therefore, identifying and targeting these clusters of malaria cases becomes imperative for a more effective and efficient malaria control [13]. This study aimed to describe epidemiological characteristics of reported malaria cases, investigate individual-level factors associated with malaria imported cases, identify indigenous malaria clusters, and explore district-level socioeconomic and environmental factors associated with a higher likelihood of malaria elimination across 154 districts in Sumatra.

## Methods

### Study setting

Sumatra is home to 21.72% of the national population, totalling 59 million people, according to the 2020 census. The island spans 480,793.28 km^2^ and has a population density of 123 people per km^2^. Administratively, Sumatra is divided into 10 provinces, 154 districts, 1,961 sub-districts, and 25,511 villages. The region is served by 2,683 primary health centres (PHCs) and 752 hospitals, with at least one PHC available in every sub-district (administrative level 3) [14].

### Study design

This study was a longitudinal retrospective study utilising individual-level national malaria surveillance data recorded in the SISMAL v2 (Malaria Information System) from 2019 to 2021. The positive cases were confirmed by microscopy or a rapid diagnostic test (RDT), which may be obtained through passive case detection (PCD) or active case detection (ACD). All *P. knowlesi* cases were reported as probable *P. knowlesi*, since polymerase chain reaction (PCR) confirmation is rarely performed, and diagnoses were based on microscopy.

### Outcome and predictor variables

For the individual-level analysis, the outcome was case classification, defined as imported and indigenous (as the reference), based on the epidemiological investigation. Cases were classified as indigenous if they had no travel history to a malaria-endemic region, and as imported if they had a travel history to a malaria-endemic region within the last 2–4 weeks. If a malaria-free district had a single case of indigenous *P. falciparum* or *P. vivax*, the district was classified as having an outbreak.

Individual-level predictor variables included age group, sex, occupation (categorised as MMP-related and non-MMP), case detection method, hospitalisation status, severity, treatment, and malaria outcome. A table summarising variables from SISMAL v2 used in the individual-level regression analysis is also available in Additional file 1: Table S1.

For the district-level analysis, the outcome was malaria elimination status in 2021, categorised as malaria-free and endemic (as the reference). District-level predictors included socioeconomic and environmental variables, such as land-use, human development index (HDI), percentage of agricultural workers, percentage of rural villages, historical Annual Parasite Incidence (API) data in 2010, and the number of neighbouring endemic districts [14–16]. HDI is a composite index calculated from life expectancy at birth, mean years of schooling, expected years of schooling, and Gross National Income (GNI) per capita [15]. Land-use data comprising the proportions of forest cover, cropland and flooded vegetation, and settlement areas [16]. A summary of the variables and their sources is available in Additional file 1: Table S2.

### Statistical analysis

The descriptive characteristics of malaria cases were analysed by summarising incidence and calculating proportions across the three-year study period. Bivariable and multivariable logistic regression analyses were conducted to explore the individual-level characteristics associated with imported malaria cases. The multivariable analysis included only cases that had undergone epidemiological investigation, enabling classification of imported and indigenous cases. The province was treated as a random effect to account for the clustering of observations within provinces.

At the district level, logistic regression was used to investigate the association between socioeconomic and environmental factors with malaria elimination status. 36 endemic and 106 malaria-free districts were included in this analysis.

Bivariable regression was first used to identify potential variables, with those with p-values < 0.10 included in the multivariable model. Multicollinearity was assessed using the Variance Inflation Factor (VIF), and variables with VIFs greater than 5 were removed. This process was repeated until all remaining variables had VIF values close to one, indicating no collinearity. Backwards stepwise selection was used to identify the most important variables, and the model with the lowest Akaike Information Criterion (AIC) was selected. The odds ratio (OR) and 95% confidence interval (CI) were summarised in a table, with p-values less than 0.05 considered statistically significant. The analysis was done using R 4.4.1.

### Missing data and sensitivity analysis

A complete case analysis was employed for individual-level data analyses. Sensitivity analysis was performed to assess the robustness of the results to missing data. After the complete case analysis was conducted, multiple imputation was applied, including all variables in the analysis model. Imputation methods for each variable were specified based on its type. Binary variables, such as occupation, hospitalisation, severity, and case detection were imputed using logistic regression method (“logreg”), while other categorical variables with more than two levels were imputed using polytomous logistic regression method (“polyreg”). A total of five imputed datasets were generated using the *mice* package [17].

### Spatial heterogeneity of indigenous malaria

The spatial relationship across the study area was first assessed using Global Moran’s *I*. Kulldorff’s spatial scan statistics were then performed using SaTScan™ v10.1.2 to identify clusters of indigenous malaria at the PHC level. Cases recorded at hospitals were reassigned to the nearest PHCs, as hospitals do not have a definitive population. Population data were collected from SISMAL v2. If the PHC did not have population data, we used the subdistrict population data from the Districts in Figures reports by the Indonesian Central Bureau of Statistics.

The analysis tested the null hypothesis that the risk of malaria incidence within a cluster was equal to that outside the cluster. The high-risk malaria clusters were detected in purely spatial settings using a circular window, with a maximum cluster size set at 50% of the population to avoid pre-selection bias and to detect both small and large clusters. Under the Poisson assumption, the likelihood ratio for each circle is calculated by:

$$LR\left(z\right)=\left\{{\left(\frac{{c}_{z}}{{n}_{z}}\right)}^{{c}_{z}}{\left(\frac{{C-c}_{z}}{{N-n}_{z}}\right)}^{C-{c}_{z}}\right\}\:I\left({c}_{z}>{n}_{z}\right)$$

where *Z* is the collection of health facilities in the study region and zone *z* consists of neighbouring geographic units with varying sizes, ${c}_{z}$ is the observed number of cases and ${n}_{z}$ is the expected number of cases. Subsequently, $C={\sum}_{z}{c}_{z}$ is the total number of cases and $N={\sum}_{z}{n}_{z}$ is the total number of expected cases. The indicator function of $I\:\left(\:\right)$ will be equal to 1 when the cluster has more cases than expected cases and 0 otherwise.

The most likely cluster, referred to as the primary cluster in this paper, was defined as the one with the maximum log-likelihood ratio (LLR), assessed at a statistical significance level of *α=0.05*, using 999 Monte Carlo replications. Once the primary cluster was identified, SaTScan removed it and re-ran the analysis to detect additional, non-overlapping clusters, referred to as secondary clusters. The Gini coefficient was used as a more systematic approach than an arbitrary number of maximum spatial window sizes to identify significant clusters [18]. Cluster map was edited using QGIS version 3.34.5.

## Results

### Epidemiological characteristics

Between 2019 and 2021, a total of 13,386 malaria cases were reported across 127 districts in Sumatra, while 27 districts reported zero cases. The highest number of cases (4,722) occurred in 2021. Most cases (88.4%) were detected in PHCs through passive case detection (83.8%) and diagnosed by microscopy (57.9%). *P. vivax* accounted for 71.8% (9,617) of the total cases, while *P. falciparum* and probable *P. knowlesi* comprised 18.4% (2,467) and 2.8% (369), respectively. Female cases accounted for 36.3%, of which 3.1% were pregnant. Nearly half of the cases (48.2%) occurred in adults aged 25–64, with a considerable proportion in young adults aged 15–24 (24.2%) and children aged under 15 (25.3%). Approximately 20% of patients required hospitalisation. Severe malaria cases and malaria-related deaths were rare, with 1% severe cases and a total of fourteen deaths. Data completeness varied across variables, with missing values ranging from 0.3% for the age group to 20.9% for completion of primaquine (PQ) medication (Table 1).

**Table 1:** Summary of malaria incidence in Sumatra region

	2019	2020	2021	Total
Number of districts reporting malaria cases	104 (67.5%)	88 (57.1%)	77 (50.0%)	127 (82.5%)
Total cases	4,467	4,197	4,722	13,386
**Demography**				
Age group				
<15	1,214 (27.2%)	1,078 (25.7%)	1,095 (23.2%)	3,387 (25.3%)
15–24	991 (22.2%)	1,051 (25.0%)	1,194 (25.3%)	3,236 (24.2%)
25–64	2,129 (47.7%)	1,979 (47.2%)	2,346 (49.7%)	6,454 (48.2%)
65+	95 (2.1%)	89 (2.1%)	87 (1.8%)	271 (2.0%)
Unknown	38 (0.9%)	0 (0%)	0 (0%)	38 (0.3%)
Gender				
Female	1,623 (36.3%)	1,569 (37.4%)	1,668 (35.3%)	4,860 (36.3%)
Male	2,842 (63.6%)	2,628 (62.6%)	3,054 (64.7%)	8,524 (63.7%)
Unknown	2 (0%)	0 (0%)	0 (0%)	2 (0%)
Pregnancy status				
Pregnant	61 (3.8%)	49 (3.1%)	41 (2.5%)	151 (3.1%)
Not pregnant	1,488 (91.7%)	1,461 (93.1%)	1,541 (92.4%)	4,490 (92.4%)
Unknown	74 (4.6%)	59 (3.8%)	86 (5.2%)	219 (4.5%)
Occupation				
Non-MMP	2,953 (66.1%)	2,777 (66.2%)	3,297 (69.8%)	9,027 (67.4%)
MMP	1,385 (31.0%)	1,346 (32.1%)	1,334 (28.3%)	4,065 (30.4%)
Unknown	129 (2.9%)	74 (1.8%)	91 (1.9%)	294 (2.2%)
**Diagnostic and treatment**				
Health facility type				
PHC	3,730 (83.5%)	3,895 (92.8%)	4,209 (89.1%)	11,834 (88.4%)
Hospital/Clinic	737 (16.5%)	302 (7.2%)	513 (10.9%)	1,552 (11.6%)
Case detection method				
ACD	554 (12.4%)	689 (16.4%)	823 (17.4%)	2,066 (15.4%)
PCD	3,859 (86.4%)	3,482 (83.0%)	3,878 (82.1%)	11,219 (83.8%)
Unknown	54 (1.2%)	26 (0.6%)	21 (0.4%)	101 (0.8%)
Diagnostic method				
Microscopy	2,952 (66.1%)	2,536 (60.4%)	2,265 (48.0%)	7,753 (57.9%)
RDT	1,492 (33.4%)	1,636 (39.0%)	2,447 (51.8%)	5,575 (41.6%)
PCR	9 (0.2%)	9 (0.2%)	1 (0%)	19 (0.1%)
Unknown	14 (0.3%)	16 (0.4%)	9 (0.2%)	39 (0.3%)
Parasite species				
* P. vivax*	2,480 (55.5%)	3,190 (76.0%)	3,947 (83.6%)	9,617 (71.8%)
* P. falciparum*	1,496 (33.5%)	564 (13.4%)	407 (8.6%)	2,467 (18.4%)
Mix	346 (7.7%)	246 (5.9%)	257 (5.4%)	849 (6.3%)
Probable *P. knowlesi*	101 (2.3%)	174 (4.1%)	94 (2.0%)	369 (2.8%)
Other	4 (0.1%)	3 (0.1%)	8 (0.2%)	15 (0.1%)
Unknown	40 (0.9%)	20 (0.5%)	9 (0.2%)	69 (0.5%)
Treatment				
Standard treatment	4,240 (94.9%)	4,024 (95.9%)	4,519 (95.7%)	12,783 (95.5%)
Non-standard treatment	7 (0.2%)	4 (0.1%)	2 (0%)	13 (0.1%)
Unknown	220 (4.9%)	169 (4.0%)	201 (4.3%)	590 (4.4%)
**Severity**				
Hospitalisation				
Inpatient	1,315 (29.4%)	671 (16.0%)	744 (15.8%)	2,730 (20.4%)
Outpatient	2,975 (66.6%)	3,484 (83.0%)	3,910 (82.8%)	10,369 (77.5%)
Unknown	177 (4.0%)	42 (1.0%)	68 (1.4%)	287 (2.1%)
Severity degree				
Severe malaria	45 (1.0%)	21 (0.5%)	64 (1.4%)	130 (1.0%)
Uncomplicated malaria	4,306 (96.4%)	4,156 (99.0%)	4,644 (98.3%)	13,106 (97.9%)
Unknown	116 (2.6%)	20 (0.5%)	14 (0.3%)	150 (1.1%)
Outcome				
Death	10 (0.2%)	2 (0%)	2 (0%)	14 (0.1%)
Recovered	4,055 (90.8%)	3,925 (93.5%)	4,574 (96.9%)	12,554 (93.8%)
Unknown	402 (9.0%)	270 (6.4%)	146 (3.1%)	818 (6.1%)
**Epidemiological investigation**				
Case classification				
Investigated	3,012 (67.4%)	3,710 (88.4%)	4,668 (98.9%)	11,390 (85.1%)
Not investigated	1,455 (32.6%)	487 (11.6%)	54 (1.1%)	1,996 (14.9%)
Source of infection				
Indigenous	1,967 (65.3%)	2,872 (77.4%)	3,909 (83.7%)	8,748 (65.4%)
Imported	694 (23.0%)	408 (11.0%)	639 (13.7%)	1,741 (13.0%)
Unknown	351 (11.7%)	430 (11.6%)	120 (2.6%)	901 (6.7%)
Malaria recurrent status ^*^				
Relapse case	154 (9.0%)	178 (6.0%)	436 (11.1%)	768 (9.0%)
New case	1,286 (75.3%)	1,598 (54.3%)	3,430 (87.3%)	6,314 (73.6%)
Unknown	267 (15.6%)	1,168 (39.7%)	62 (1.6%)	1,497 (17.4%)
PQ completion ^†^				
Complete	1,146 (55.8%)	2,876 (90.2%)	3,216 (76.8%)	7,238 (76.8%)
Incomplete	52 (2.5%)	11 (0.3%)	17 (0.4%)	80 (0.8%)
Unobserved	104 (5.1%)	32 (1.0%)	7 (0.2%)	143 (1.5%)
Unknown	751 (36.6%)	271 (8.5%)	945 (22.6%)	1,967 (20.9%)
Suspected origin				
Sumatera	168 (24.2%)	148 (36.3%)	348 (54.5%)	664 (38.1%)
Java & Bali	3 (0.4%)	0 (0%)	0 (0%)	3 (0.2%)
Papua	209 (30.1%)	18 (4.4%)	66 (10.3%)	293 (16.8%)
Unknown	314 (45.2%)	242 (59.3%)	225 (35.2%)	781 (44.9%)

Data in n (%)

*MMP* Mobile and migrant population, *PHC* Primary health centre, *ACD* Active case detection, *PCD* Passive case detection, *RDT* Rapid diagnostic test, *PCR* Polymerase chain reaction, *PQ* Primaquine

^*^ Malaria recurrent status was defined from the investigated *P. vivax* cases

^†^ PQ medication was considered complete if the patients take all PQ for 14-days. PQ completions were calculated from investigated *P. vivax* and Mix cases

A total of 11,390 cases (85.1%) were investigated, of which 8,748 (76.8%) were classified as indigenous, 1,741 (15.3%) as imported, and 901 (7.9%) remained unclassified. Interestingly, indigenous cases were reported in 27 malaria-free districts, suggesting the presence of outbreaks. Rokan Hilir district in Riau province recorded the highest number of outbreak cases, accounting for 20% of all cases (2,675/13,386). The outbreaks peaked in June and October 2020.

Results from the complete-case analysis were consistent in direction and magnitude with those from multiple imputation, suggesting that missing data did not substantially influence the findings. Comparison of the results is presented in Additional file 1: Table S3. Indigenous and imported cases exhibited distinct epidemiological characteristics, as indicated by logistic regression analyses (*N* = 10,163), except for treatment received and malaria outcomes (Table 2). After adjusting for other variables, imported cases were associated with working age groups (adjusted odds ratio [aOR] 1.79, 95% CI 1.42–2.25 for 15–24 years old, and 1.97, 1.59–2.46 for 25–64 years old, compared to < 15 years old), being male (aOR 1.70, 95% CI 1.43–2.03), working in MMP-related occupation (aOR 1.46, 95% CI 1.22–1.76), diagnosed at hospitals or clinics (aOR 2.32, 95% CI 1.85–2.91), infected with *P. falciparum* (aOR 1.48, 95% CI 1.22–1.80), and being hospitalised (aOR 15.29, 95% CI 12.66–18.46).

**Table 2:** Comparison of characteristics between imported and indigenous cases (*N* = 10,163)

Variable	Imported	Indigenous	Unadjusted OR (95% CI)	*p*-value	Adjusted OR (95% CI)	*p*-value
Age group						
<15	190 (11.6%)	2,394 (28.1%)	Ref		Ref	
15–24	404 (24.7%)	2,140 (25.1%)	2.52 (2.08–3.05)	< 0.001	1.79 (1.42–2.25)	< 0.001
25–64	1,000 (61.2%)	3,831 (44.9%)	3.62 (3.05–4.30)	< 0.001	1.97 (1.59–2.46)	< 0.001
65+	39 (2.4%)	165 (1.9%)	3.24 (2.17–4.83)	< 0.001	1.29 (0.79–2.11)	0.317
Gender						
Female	393 (24.1%)	3,304 (38.7%)	Ref		Ref	
Male	1,240 (75.9%)	5,226 (61.3%)	2.15 (1.89–2.44)	< 0.001	1.70 (1.43–2.03)	< 0.001
Occupation						
Non-MMP	966 (59.2%)	6,096 (71.5%)	Ref		Ref	
MMP	667 (40.8%)	2,434 (28.5%)	1.98 (1.76–2.24)	< 0.001	1.46 (1.22–1.76)	< 0.001
Health facility type						
PHC	1,053 (64.5%)	8,060 (94.5%)	Ref		Ref	
Hospital/Clinic	580 (35.5%)	470 (5.5%)	15.62 (13.2-18.47)	< 0.001	2.32 (1.85–2.91)	< 0.001
Case detection method						
PCD	1,490 (91.2%)	6,951 (81.5%)	Ref		Ref	
ACD	143 (8.8%)	1,579 (18.5%)	0.37 (0.30–0.45)	< 0.001	0.77 (0.62–0.96)	0.021
Diagnostic method						
Microscopy	1,211 (74.2%)	4,742 (55.6%)	Ref		Ref	
RDT	417 (25.5%)	3,774 (44.2%)	0.48 (0.42–0.54)	< 0.001	1.07 (0.90–1.26)	0.448
PCR	5 (0.3%)	14 (0.2%)	2.57 (0.83–7.96)	0.102	2.8 (0.65–12.04)	0.167
Parasite						
* P. vivax*	1,105 (67.7%)	6,696 (78.5%)	Ref		Ref	
* P. falciparum*	412 (25.2%)	1,026 (12.0%)	1.70 (1.45-2.00)	< 0.001	1.48 (1.22–1.80)	< 0.001
Mix	92 (5.6%)	508 (6.0%)	0.92 (0.72–1.17)	0.493	0.87 (0.64–1.20)	0.395
Probable *P. knowlesi*	19 (1.2%)	295 (3.5%)	0.68 (0.37–1.26)	0.221	0.10 (0.05–0.20)	< 0.001
Other	5 (0.3%)	5 (0.1%)	5.15 (1.41–18.79)	0.013	5.56 (1.05–29.37)	0.044
Hospitalisation						
Outpatient	509 (31.2%)	7,625 (89.4%)	Ref		Ref	
Inpatient	1,124 (68.8%)	905 (10.6%)	24.22 (20.93–28.03)	< 0.001	15.29 (12.66–18.46)	< 0.001
Severity degree						
Uncomplicated malaria	1,606 (98.3%)	8,463 (99.2%)	Ref		Ref	
Severe malaria	27 (1.7%)	67 (0.8%)	1.75 (1.07–2.87)	0.026	1.06 (0.53–2.12)	0.876
Treatment						
Standard treatment	1,531 (99.8%)	8,267 (99.9%)	Ref			
Non-standard treatment	3 (0.2%)	5 (0.1%)	1.94 (0.27–13.82)	0.507		
Outcome						
Recovered	1,549 (99.7%)	8,188 (99.8%)	Ref			
Death	4 (0.3%)	9 (0.1%)	1.17 (0.28–4.79)	0.829		

Data on imported and indigenous are in n (%)

*OR* Odds ratio, *CI* Confidence interval, *MMP* Mobile and migrant population, *PHC* Primary health centre, *ACD* Active case detection, *PCD *Passive case detection, *RDT *Rapid diagnostic test, *PCR* Polymerase chain reaction

**Fig. 1:**
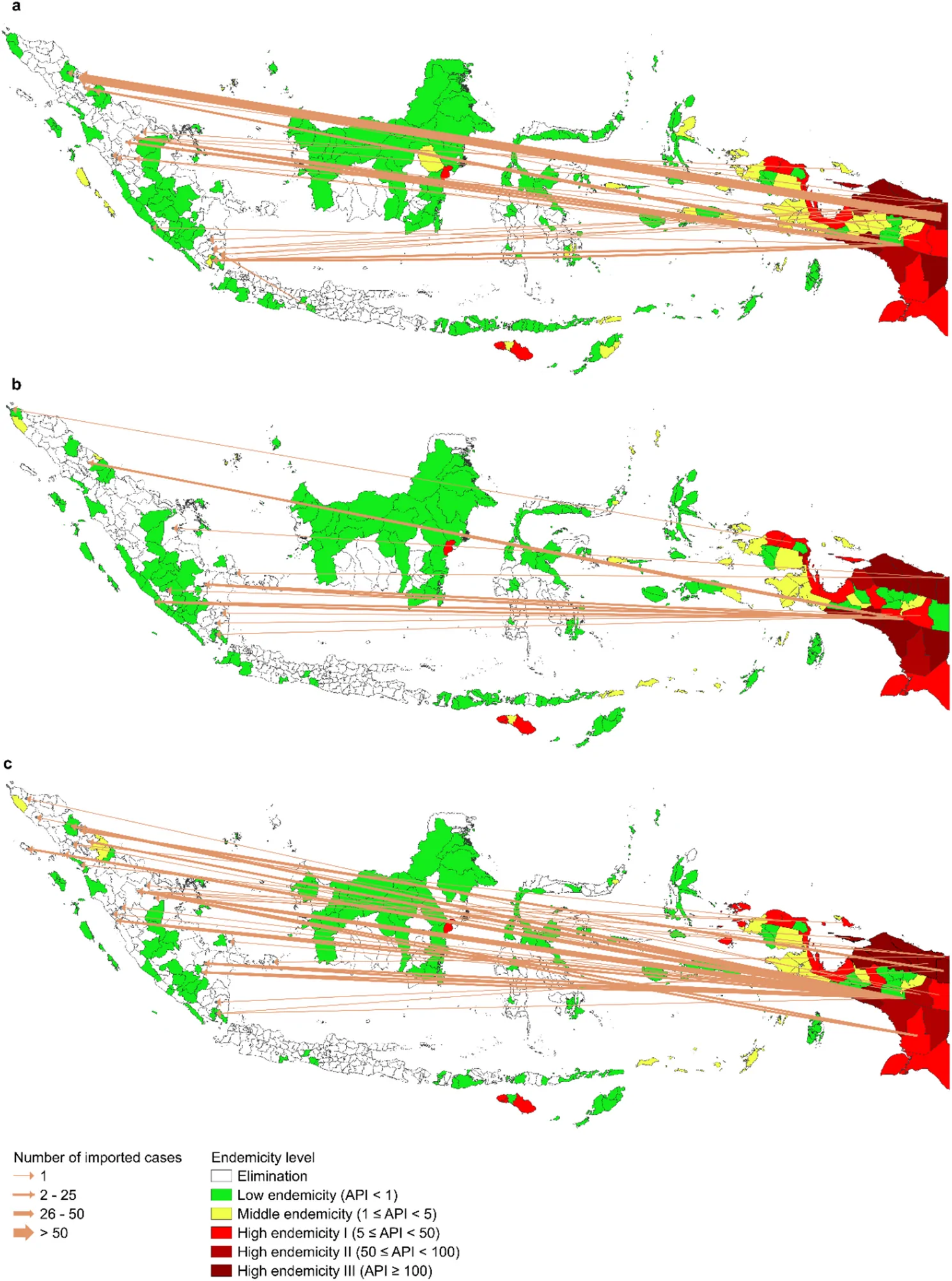
Flow map of imported malaria cases from outside Sumatra region, (**a**) 2019, (**b**) 2020, (**c**) 2021. *API*  Annual parasite incidence. Base map illustrates the district endemicity. Origin and destination points represent the district centroid. Thickness of the line represents the number of malaria cases imported from the suspected origin

**Fig. 2:**
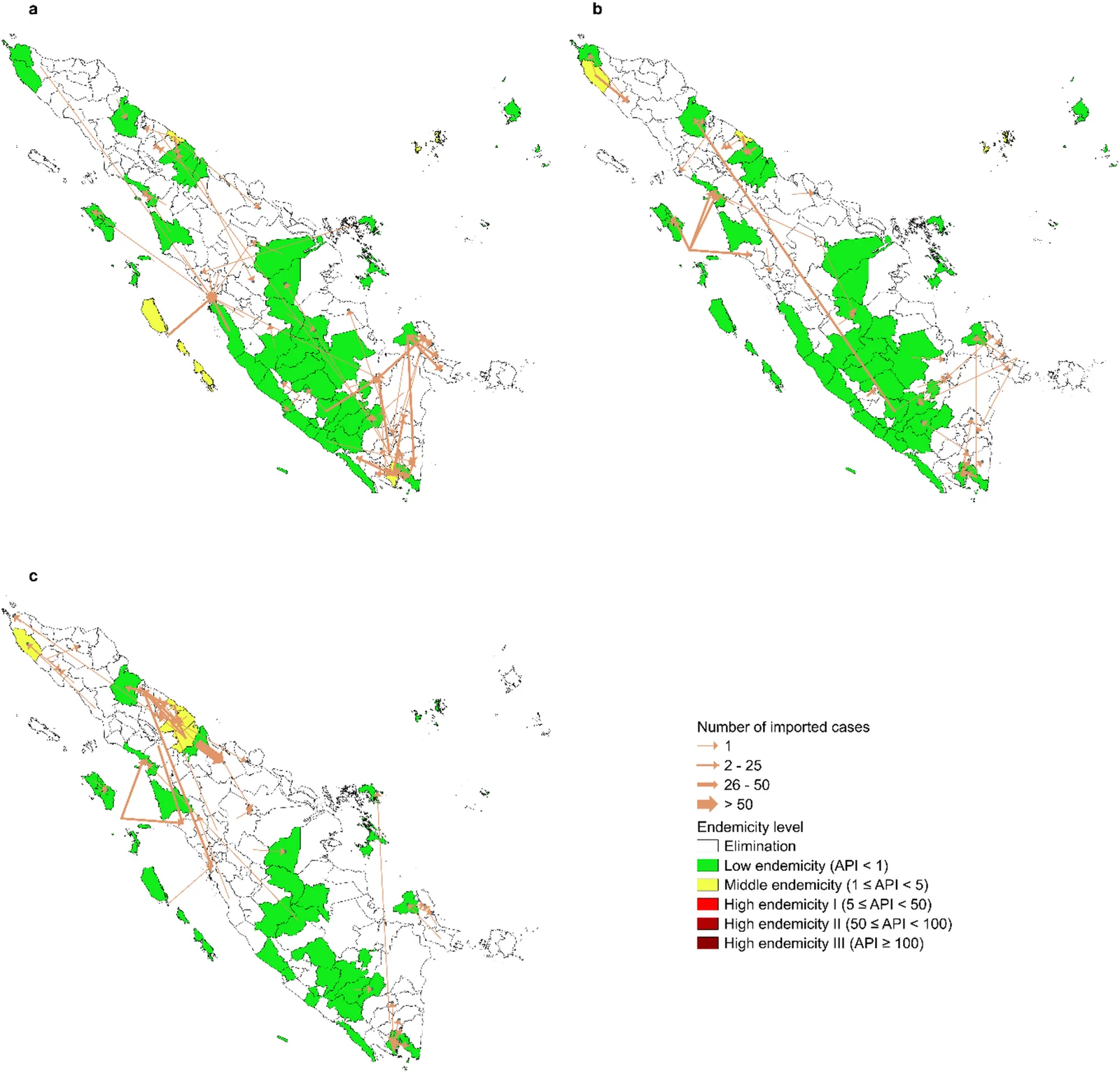
Flow map of imported malaria cases from within Sumatra region, (**a**) 2019, (**b**) 2020, (**c**) 2021. *API*  Annual parasite incidence. Base map illustrates the district endemicity. Origin and destination points represent the district centroid. Thickness of the line represents the number of malaria cases imported from the suspected origin

We were able to determine the suspected origin of the importation for around half of the investigated cases (960, 55.1%). Most of the importations originated from other districts in Sumatra (38.1%) and Papua (16.8%). Almost half of the imported cases (48.6%) came from low-endemic districts, and around 20% were from very high-endemic districts (API ≥ 100). Notably, 93 imported cases (9.7%) originated from malaria-free districts. Flow maps indicated that the movement of imported cases, both from outside (Fig. 1) and within (Fig. 2) Sumatra, fluctuated annually, with 2020 being the lowest due to COVID-19 mobility restrictions.

### District-level factors associated with malaria elimination

Multivariable logistic regression shows that the human development index, API in 2010, and the number of neighbouring endemic districts were significantly associated with districts achieving malaria elimination (Table 3). The higher the HDI, the more likely the district is to eliminate malaria (aOR 1.15, 95% CI 1.01–1.34). On the contrary, a higher historical API in 2010 reduced the likelihood of eliminating malaria (aOR 0.46, 95% CI 0.29–0.69). Similarly, a higher number of neighbouring endemic districts decreased the likelihood of malaria elimination (aOR 0.78, 95% CI 0.67–0.89).

**Table 3:** Socioeconomic and environmental factors associated with malaria elimination

	Unadjusted OR (95% CI)	*p*-value	Adjusted OR (95% CI)	*p*-value
% Forest	0.96 (0.93–0.98)	0.001		
% Cropland and flooded vegetation	1.06 (1.02–1.12)	0.013		
% Settlement	1.05 (1.01–1.12)	0.033		
% Agriculture workers	0.97 (0.95–0.99)	0.014		
% Rural village	0.97 (0.95–0.99)	0.003		
Human development index	1.19 (1.08–1.34)	0.001	1.15 (1.01–1.34)	0.042
API in 2010	0.79 (0.68–0.89)	<0.0001	0.78 (0.67–0.89)	<0.0001
Number of endemic district neighbour	0.50 (0.35–0.70)	<0.0001	0.46 (0.29–0.69)	<0.0001

*OR* Odds ratio, *CI* Confidence interval, *API* Annual parasite index

### Spatial heterogeneity of indigenous malaria

**Fig. 3:**
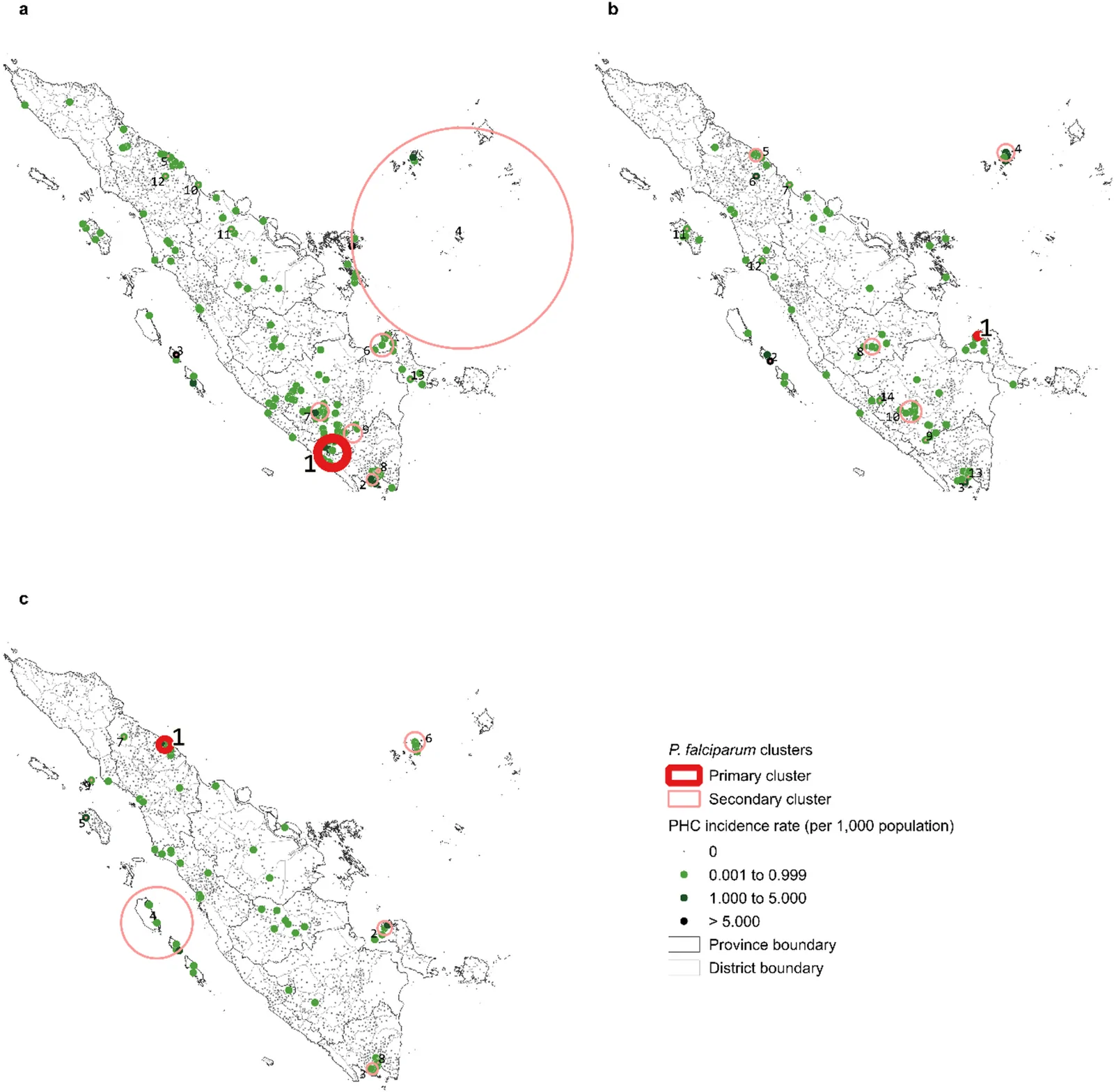
Clusters of P. *falciparum* in (**a**) 2019, (**b**) 2020, (**c**) 2021. Red circles represent primary cluster, and pink circles represent secondary cluster. Point symbol represents primary health care location and the darker colour represents the higher incidence rate (per 1,000 population)

**Fig. 4:**
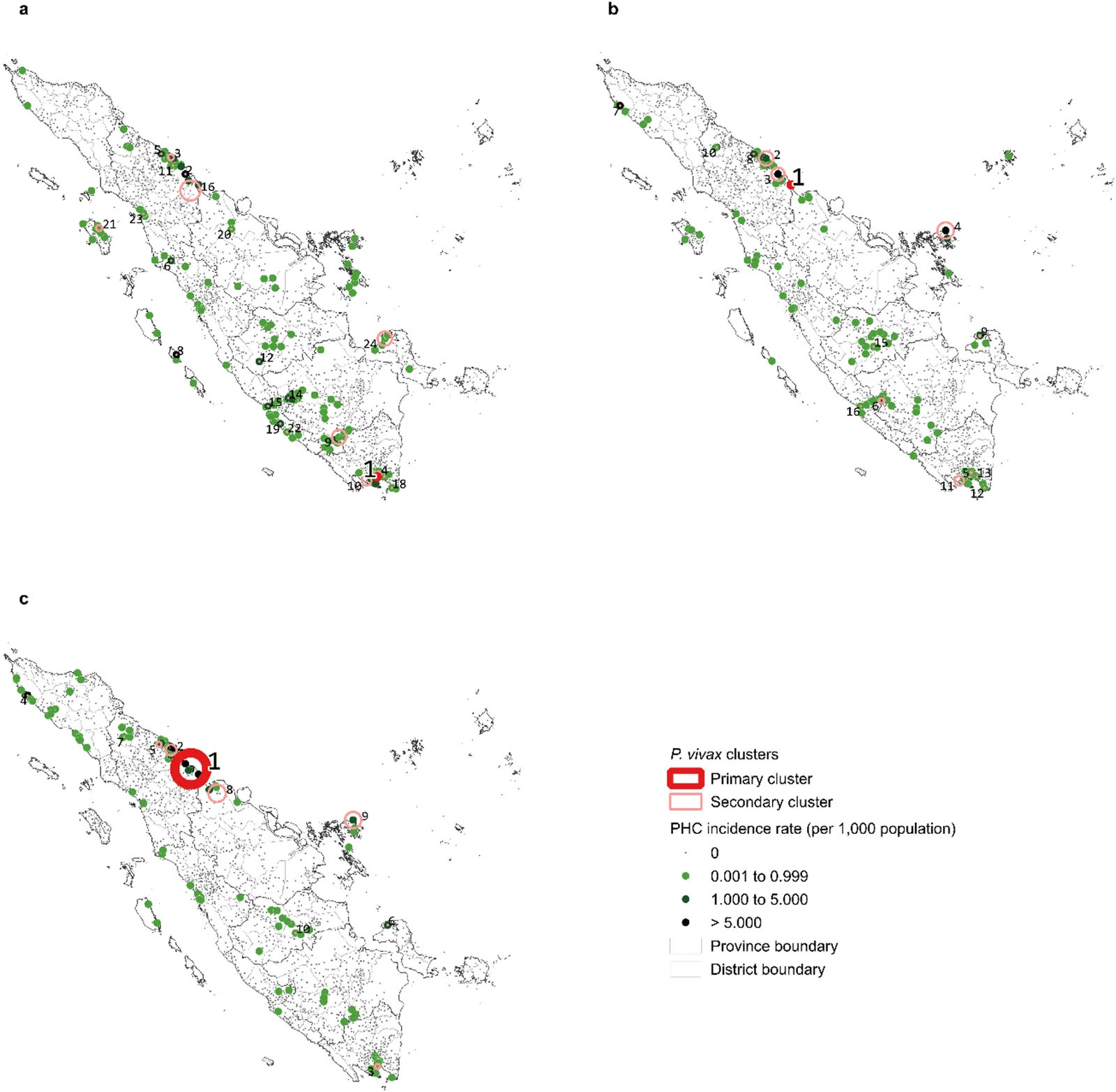
Clusters of *P. vivax* in (**a**) 2019, (**b**) 2020, (**c**) 2021. Red circles represent primary cluster and pink circles represent secondary cluster. Point symbol represents primary health care location and the darker colour represents the higher incidence rate (per 1,000 population)

The number of PHCs used for the indigenous cluster analysis was 2,460, with 9,271 cases, representing 69% of the total cases in Sumatra from 71 districts. Spatial autocorrelation analysis using Global Moran’s *I* showed significant clustering of malaria cases across the region (Moran’s *I* = 0.285, z-score = 4.485, p-value < 0.001). The primary clusters of indigenous malaria cases were detected in different provinces each year for each *P. falciparum* and *P. vivax* (Figs. 3 and 4, respectively), except for the probable *P. knowlesi* cluster (Additional file 1: Fig. S1), which was confined to Aceh province. No overlapping clusters were observed between species. Several secondary clusters were also detected, with *P. vivax* having the largest number of clusters (Additional file 1: Tables S2-S3).

The Hanura PHC in Lampung province was consistently detected as a *P. vivax* cluster centre for three years. In 2019, it was a primary cluster with an estimated relative risk of 333.4 covering a radius of less than 1 km and a population of 37,400. While in the following years, it was detected as a secondary cluster. In 2020, the primary cluster for *P. vivax* was centred at Panipahan PHC in Riau province, with a radius of less than 1 km and a population of 38,402. The estimated risk of malaria within this cluster was 1,563.7 times higher compared to the surrounding areas, indicating that the outbreak in the Rokan Hilir district was concentrated in this PHC catchment area. In 2021, the cluster centre shifted to the neighbouring Sei Berombang PHC in North Sumatra province, which had a radius of 48.9 km and a population of 418,029. The estimated relative risk of this cluster was 111.1.

## Discussion

This study is among the first to investigate the epidemiology of malaria and district-level socioeconomic and environmental factors associated with the likelihood of malaria elimination across 154 districts in Sumatra, Indonesia. Our analysis revealed that while *P. vivax* infections accounted for the majority of malaria cases, *P. falciparum* remained prevalent, and a substantial proportion of probable *P. knowlesi* infections was also reported. Around 15% of the total reported cases were imported, contributing to the reintroduction of malaria transmission in areas that had already achieved or were nearing elimination. Compared to indigenous cases, imported cases were more likely to involve males of working age, working in MMP-related occupations, diagnosed at hospitals or clinics, infected with *P. falciparum*, and being hospitalised. At the district level, higher HDI, lower historical API, and fewer endemic district neighbours were associated with a higher likelihood of eliminating malaria. Furthermore, our spatial analysis indicated a clustering tendency, with various localised malaria clusters identified for *P. falciparum*, *P. vivax*, and probable *P. knowlesi*.

The demographic characteristics of malaria cases in Sumatra were predominantly males aged 15–64 years. Several studies have observed this epidemiological shift towards increasing cases among adults and males as the region progresses towards the pre-elimination phase [12]. Our individual-level analysis further revealed that imported malaria cases were strongly associated with MMP-related occupations, consistent with findings from other Southeast Asia settings, including Cambodia [19] and Malaysia [20]. Imported cases were also reported originating from malaria-free areas, which may reflect challenges in accurately determining transmission origin in the context of high population mobility between endemic and malaria-free districts. In addition, imported *P. vivax* cases in malaria-free districts may pose a risk of reintroduction if undetected or inadequately treated [21]. Mandatory screening for MMPs before re-entering malaria-free areas needs to be reinforced to prevent reintroduction and resurgence of local transmission.

Outbreaks during 2019–2021 in malaria-free districts further highlight the need to maintain control and elimination efforts. Investigations by the NMP and independent experts have identified the potential source of transmission that caused the outbreak in Rokan Hilir in 2020: an imported case involving fishermen who had stayed overnight in an endemic area. There were also several factors contributing to the outbreaks, including delays in response and reporting, logistical challenges in case detection, and issues in program management [22]. Additionally, the Indonesian government imposed mobility restrictions and shifted healthcare priorities during COVID-19 [23], which reduced the ability of healthcare workers to conduct outbreak control measures and routine case detection. Consequently, there was a decline in malaria testing and reporting, as well as delays in the distribution of long-lasting insecticide-treated nets (LLINs) and in indoor residual spraying (IRS) activity [6]. A mathematical modelling study investigated that the greatest COVID-19 impact on malaria in low- and middle-income countries was the interruption of LLINs distribution, which can lead to a 36% increase in deaths due to malaria [24].

Spatial analysis results showed that indigenous malaria incidence in the Sumatra region tended to be clustered in close neighbourhoods. SaTScan detected several localised clusters, with distinct cluster centres for *P. falciparum* and *P. vivax* each year, indicating temporal instability in cluster locations. This instability could be due to the human movement [25] and ecological factors, such as proximity to breeding sites [26]. For example, in 2021, the *P. vivax* cluster centre was shifted from the outbreak location in Riau province to the neighbouring endemic area of North Sumatra province. This spatial pattern suggests that the outbreak may have been driven by imported cases originating from the neighbouring area. By contrast, a cluster in Lampung province persisted throughout the study period, indicating that local sociodemographic and environmental factors may sustain the ongoing transmission [27]. The stability of this cluster also suggests it as a prime location for targeted control interventions. These dynamics highlight the importance of strong surveillance to detect, report, and respond to importation cases early, while also maintaining targeted interventions in endemic areas to reduce the source of importation and prevent reintroduction in malaria-free areas.

Our district-level analysis suggests that malaria elimination was more likely to be associated with higher HDI, a lower history of API over the last decade, and fewer neighbouring endemic districts. Indonesia NMP adopts the WHO definition of elimination, which requires three consecutive years without indigenous cases, highlighting the importance of strong health system capacity to sustain malaria control and elimination efforts. As suggested by Cohen et al., the median time from the implementation of effective control measures to malaria elimination was 12 years [28]. This finding further indicates that malaria-free status is vulnerable to its neighbouring epidemiological situation, as human mobility crosses administrative borders. This suggests the need for robust cross-border surveillance, which is one of the key interventions to reduce malaria incidence [29].

Parasite-specific challenges further complicate efforts to eliminate parasites in Sumatra. *P. vivax* remains the most difficult species to eliminate due to its ability to relapse and poor adherence to PQ treatment. Completion rates were as low as 33% in malaria-free districts. This situation highlights the need to improve adherence, for example, by introducing tafenoquine (TQ) when glucose-6-phosphate-dehydrogenase (G6PD) testing is available, as it offers a shorter treatment duration [30].

Meanwhile, *P. knowlesi* is difficult to diagnose using microscopy because of its morphological similarity to other species, and confirmation requires PCR. However, PCR capacity is limited to a few centres in large cities [31]. The ability of Aceh province to diagnose probable *P. knowlesi* cases is largely due to the presence of expert-level (L1) microscopists who have been specifically trained to recognise *P. knowlesi* morphology in blood smears. Investing in PCR testing is therefore recommended to accurately assess the burden of *P. knowlesi* in areas identified as high-risk for *P. knowlesi* transmission, such as Aceh and North Sumatra province [32]. In addition, strengthening microscopy capacity in other areas is important to reduce misdiagnosis and inaccuracy of parasite species identification. Accurate diagnosis and parasite identification are necessary for accurate treatment and successful disease elimination [33, 34].

Several limitations should be considered when interpreting the findings of this study. First, malaria cases reported by SISMAL may suffer from underreporting, which may result in an underestimated burden of malaria in this study. Therefore, improved quality of surveillance data will enable better estimation of malaria burden in the future. Secondly, the analysis of district-level association was limited to a few socioeconomic and environmental factors, which may not fully account for potential confounders such as altitude, distance from forest, and rainfall [11]. Finally, the results from SaTScan should be interpreted with caution, as sparse data distributed across large areas may produce inflated relative risk estimates [35]. While this does not invalidate the location of the clusters, it reduces the reliability of relative risk values. Aggregating data to a coarser level, such as by district, could mitigate this issue, although it risks losing the finer details needed to identify localised clusters within districts.

## Conclusions

In conclusion, this study demonstrated that malaria cases in Sumatra share characteristics with those observed in low- to moderate-transmission settings. The considerable proportion of imported cases, particularly among working-age males engaged in MMP-related occupations, poses a significant threat to elimination efforts. This is particularly true for malaria-endemic districts with a lower human development index, higher historical API, and a higher number of neighbouring endemic districts. These findings highlight the need for strengthened surveillance and targeted interventions tailored to local transmission settings, including improved case detection and diagnosis, especially for *P. knowlesi*, cross-border surveillance, and comprehensive epidemiological investigations. Furthermore, sustained collaboration among health authorities, local stakeholders, and vulnerable communities is essential to prevent reintroduction, control outbreaks, and advance malaria elimination.

## Supplementary Information

Below is the link to the electronic supplementary material.

Supplementary Material 1: Additional file 1: Fig. S1 and Tables S1–S6.

## Data Availability

The malaria data that support the findings of this study are from the Malaria Working Group, Ministry of Health of Indonesia, but restrictions apply to the availability of these data, which were used under license for the current study, and so are not publicly available. Data are however available upon reasonable request and with permission of the Malaria Working Group, Ministry of Health of Indonesia. The socioeconomic and environmental variables are available in the Indonesia Central Bureau of Statistics repository (https://www.bps.go.id/en/publication/2022/02/25/0a2afea4fab72a5d052cb315/statistical-yearbook-of-indonesia-2022.html, https://www.bps.go.id/id/publication/2022/05/11/48b6466dcf14b562df9f17e2/indeks-pembangunan-manusia-2021.html) and ESRI repository (https://livingatlas.arcgis.com/landcoverexplorer/). Reproducible code for data analysis and visualisation is available at https://github.com/karinadnlstr/malaria-sumatra.

## Abbreviations

ACD: Active case detection
AIC: Akaike information criterion
aOR: Adjusted odds ratio
API: Annual Parasite Incidence
CI: Confidence interval
G6PD: Glucose-6-phosphate-dehydrogenase
GNI: Gross National Income
HDI: Human development index
IRS: Indoor residual spraying
LLIN: Long-lasting insecticidal net
LLR: Log-likelihood ratio
MMP: Mobile and migrant population
NMP: National Malaria Program
OR: Odds ratio
PCD: Passive case detection
PCR: Polymerase chain reaction
PHC: Primary health centre
PQ: Primaquine
RDT: Rapid diagnostic test
SISMAL: Sistem Informasi Malaria or Malaria Information System
TQ: Tafenoquine
VIF: Variance Inflation Factor
WHO: World Health Organisation
