# Supplementary material for: Epidemiology of malaria and district-level factors associated with malaria elimination in Sumatra region, Indonesia: a retrospective analysis of surveillance data
Source: BMC Med. 2026 Jul 29;24:592. doi: 10.1186/s12916-026-05104-3 (PMC13711844; doi:10.1186/s12916-026-05104-3)
Supplement: Supplementary file 1 [file 12916_2026_5104_MOESM1_ESM.docx]

**Additional file 1**

**Epidemiology of malaria and district-level factors associated with malaria elimination in Sumatra region, Indonesia: a retrospective analysis of surveillance data**

Karina D Lestari, Henry Surendra, Bimandra A Djaafara, Ihsan Fadilah, Lenny L Ekawati, Hermawan Susanto, Hellen D Prameswari, Sri B Fajariyani, Dedy Supriyanto, Herdiana Herdiana, Eamon Conway, Pimphen Charoen, Saranath Lawpoolsri, Wirichada Pan-ngum, Iqbal R F Elyazar

**List of tables and figures**

[Table S1 Summary of SISMAL v2 variables included in individual-level logistic regression with case classification as the outcome variable and indigenous cases category as the reference.	2](#_Toc227261155)

[Table S2 Summary of explanatory variables included in district-level logistic regression with malaria elimination status as the outcome variable and endemic districts as the reference.	2](#_Toc227261156)

[Table S3 Comparison of the results between complete case analysis and multiple imputation.	3](#_Toc227261157)

[Table S4 Summary of *P. falciparum* clusters by year and province.	4](#_Toc227261158)

[Table S5 Summary of *P. vivax* clusters by year and province.	6](#_Toc227261159)

[Table S6 Summary of probable *P. knowlesi* clusters by year and province.	8](#_Toc227261160)

[Figure S1 Clusters of probable *P. knowlesi* in a) 2019, b) 2020, c) 2021. Red circles represent the primary cluster, and pink circles represent the secondary cluster. The point symbol represents the primary health care location, and the darker colour represents the higher incidence rate (per 1,000 population).	9](#_Toc227261162)

Table S1 Summary of SISMAL v2 variables included in individual-level logistic regression with case classification as the outcome variable and indigenous cases category as the reference.

|  | **Variable** |
| --- | --- |
| Demographic | Age group |
|  | Gender |
|  | Pregnancy status |
|  | Occupation |
| Diagnostic and treatment | Health facility type |
|  | Case finding method |
|  | Diagnostic method |
|  | Parasite species |
|  | Treatment |
| Severity | Severity degree |
|  | Hospitalisation status |
|  | Outcome |
| Epidemiological investigation^†^ | Case classification |
|  | Source of infection |
|  | Malaria recurrent status |
|  | Primaquine 14-day completion |
|  | Suspected location of infection |

^†^ Variables in the epidemiological investigation were only used to summarise the characteristics of imported cases and were not included in the regression analysis.

Table S2 Summary of explanatory variables included in district-level logistic regression with malaria elimination status as the outcome variable and endemic districts as the reference.

| **Variable** | **Data source** |
| --- | --- |
| Human development index (HDI) | Indonesia Central Bureau of Statistics [1, 2] |
| Percentage of agricultural workers |  |
| Percentage of rural village data |  |
| Annual Parasite Incidence (API) | SISMAL v1 |
| Percentage of forest cover | ESA Sentinel-2 Satellite at 10-meter resolution and aggregated to the district level [3] |
| Percentage of cropland and flooded vegetation |  |
| Percentage of settlement areas |  |
| Number of neighbouring endemic districts | Calculated based on the district adjacency matrix |

Table S3 Comparison of the results between complete case analysis and multiple imputation.

| **Variable** | **Imported** | **Indigenous** | **Complete case** | | **Multiple imputation** | |
| --- | --- | --- | --- | --- | --- | --- |
|  |  |  | **Adjusted OR (95% CI)** | **p-value** | **Adjusted OR (95% CI)** | **p-value** |
| Age group |  |  |  |  |  |  |
| < 15 | 190 (11.6) | 2,394 (28.1) | Ref |  |  |  |
| 15-24 | 404 (24.7) | 2,140 (25.1) | 1.79 (1.42-2.25) | <0.001 | 1.77 (1.41-2.23) | <0.001 |
| 25-64 | 1,000 (61.2) | 3,831 (44.9) | 1.97 (1.59-2.46) | <0.001 | 1.96 (1.58-2.44) | <0.001 |
| 65+ | 39 (2.4) | 165 (1.9) | 1.29 (0.79-2.11) | 0.317 | 1.26 (0.77-2.07) | 0.355 |
| Gender |  |  |  |  |  |  |
| Female | 393 (24.1) | 3,304 (38.7) | Ref |  |  |  |
| Male | 1,240 (75.9) | 5,226 (61.3) | 1.7 (1.43-2.03) | <0.001 | 1.68 (1.42-1.99) | <0.001 |
| Occupation |  |  |  |  |  |  |
| Non-MMP | 966 (59.2) | 6,096 (71.5) | Ref |  |  |  |
| MMP | 667 (40.8) | 2,434 (28.5) | 1.46 (1.22-1.76) | <0.001 | 1.47 (1.23-1.77) | <0.001 |
| Health facility type |  |  |  |  |  |  |
| PHC | 1,053 (64.5) | 8,060 (94.5) | Ref |  |  |  |
| Hospital/Clinic | 580 (35.5) | 470 (5.5) | 2.32 (1.85-2.91) | <0.001 | 2.37 (1.89-2.96) | <0.001 |
| Case detection method |  |  |  |  |  |  |
| PCD | 1,490 (91.2) | 6,951 (81.5) | Ref |  |  |  |
| ACD | 143 (8.8) | 1,579 (18.5) | 0.77 (0.62-0.96) | 0.021 | 0.77 (0.62-0.97) | 0.023 |
| Diagnostic method |  |  |  |  |  |  |
| Microscopy | 1,211 (74.2) | 4,742 (55.6) | Ref |  |  |  |
| RDT | 417 (25.5) | 3,774 (44.2) | 1.07 (0.9-1.26) | 0.448 | 1.03 (0.88-1.22) | 0.69 |
| PCR | 5 (0.3) | 14 (0.2) | 2.8 (0.65-12.04) | 0.167 | 2.74 (0.64-11.72) | 0.175 |
| Parasite |  |  |  |  |  |  |
| Pv | 1,105 (67.7) | 6,696 (78.5) | Ref |  |  |  |
| Pf | 412 (25.2) | 1,026 (12.0) | 1.48 (1.22-1.8) | <0.001 | 1.42 (1.17-1.73) | <0.001 |
| Mix | 92 (5.6) | 508 (6.0) | 0.87 (0.64-1.2) | 0.395 | 0.85 (0.62-1.16) | 0.311 |
| Suspect Pk | 19 (1.2) | 295 (3.5) | 0.1 (0.05-0.2) | <0.001 | 0.1 (0.05-0.2) | <0.001 |
| Other | 5 (0.3) | 5 (0.1) | 5.56 (1.05-29.37) | 0.044 | 5.44 (1.02-29.02) | 0.047 |
| Hospitalisation |  |  |  |  |  |  |
| Outpatient | 509 (31.2) | 7,625 (89.4) | Ref |  |  |  |
| Inpatient | 1,124 (68.8) | 905 (10.6) | 15.29 (12.66-18.46) | <0.001 | 15.03 (12.46-18.13) | <0.001 |
| Severity degree |  |  |  |  |  |  |
| Uncomplicated malaria | 1,606 (98.3) | 8,463 (99.2) | Ref |  |  |  |
| Severe malaria | 27 (1.7) | 67 (0.8) | 1.06 (0.53-2.12) | 0.876 | 1.09 (0.55-2.16) | 0.801 |

Table S4 Summary of *P. falciparum* clusters by year and province.

| **Year** | **Cluster** | **Province** | **Radius (km)** | **No of PHC** | **Pop** | **No of cases** | **% case** | **RR** | **p-value** |
| --- | --- | --- | --- | --- | --- | --- | --- | --- | --- |
| **2019** | 1 | South Sumatra | 44.8 | 25 | 461,528 | 309 | 22.6 | 32.1 | < 0.001 |
|  | 2 | Lampung | 17.0 | 7 | 154,647 | 233 | 17.1 | 67.8 | < 0.001 |
|  | 3 | West Sumatra | < 1 | 1 | 7,454 | 117 | 8.6 | 642.7 | < 0.001 |
|  | 4 | Riau Island | 336.2 | 26 | 178,506 | 104 | 7.6 | 23.5 | < 0.001 |
|  | 5 | North Sumatra | < 1 | 1 | 33,025 | 57 | 4.2 | 67.4 | < 0.001 |
|  | 6 | Bangka Belitung Island | 34.3 | 7 | 164,374 | 68 | 5 | 16.2 | < 0.001 |
|  | 7 | South Sumatra | 26.6 | 19 | 421,806 | 91 | 6.7 | 8.6 | < 0.001 |
|  | 8 | Lampung | 5.0 | 15 | 505,526 | 63 | 4.6 | 4.8 | < 0.001 |
|  | 9 | Lampung | 27.4 | 18 | 408,694 | 55 | 4 | 5.2 | < 0.001 |
|  | 10 | Riau | < 1 | 1 | 38,402 | 20 | 1.5 | 19.8 | < 0.001 |
|  | 11 | Riau | < 1 | 1 | 22,550 | 16 | 1.2 | 26.9 | < 0.001 |
|  | 12 | North Sumatra | < 1 | 1 | 21,819 | 14 | 1 | 24.3 | < 0.001 |
|  | 13 | Bangka Belitung Island | < 1 | 1 | 8,336 | 7 | 0.5 | 31.6 | < 0.001 |
| **2020** | 1 | Bangka Belitung Island | 1.8 | 2 | 33,306 | 105 | 19.5 | 375.0 | < 0.001 |
|  | 2 | West Sumatra | < 1 | 1 | 2,423 | 37 | 6.9 | 1570.8 | < 0.001 |
|  | 3 | Lampung | < 1 | 1 | 13,645 | 41 | 7.6 | 311.5 | < 0.001 |
|  | 4 | Riau Island | 25.7 | 4 | 29,857 | 38 | 7.1 | 131.1 | < 0.001 |
|  | 5 | North Sumatra | 19.0 | 14 | 377,376 | 66 | 12.3 | 19.0 | < 0.001 |
|  | 6 | North Sumatra | < 1 | 1 | 21,819 | 27 | 5 | 124.8 | < 0.001 |
|  | 7 | Riau | < 1 | 1 | 38,402 | 29 | 5.4 | 76.4 | < 0.001 |
|  | 8 | Jambi | 24.0 | 4 | 97,686 | 19 | 3.5 | 19.3 | < 0.001 |
|  | 9 | South Sumatra | < 1 | 1 | 42,128 | 10 | 1.9 | 23.2 | < 0.001 |
|  | 10 | South Sumatra | 32.5 | 23 | 478,187 | 23 | 4.3 | 4.8 | < 0.001 |
|  | 11 | North Sumatra | < 1 | 1 | 10,141 | 5 | 0.9 | 47.7 | < 0.001 |
|  | 12 | West Sumatra | < 1 | 1 | 37,586 | 7 | 1.3 | 18.1 | 0.001 |
|  | 13 | Lampung | < 1 | 1 | 37,739 | 7 | 1.3 | 18.0 | 0.001 |
|  | 14 | Bengkulu | < 1 | 1 | 12,405 | 5 | 0.9 | 39.0 | 0.002 |
| **2021** | 1 | North Sumatra | 19.0 | 14 | 378,872 | 116 | 33.5 | 68.1 | < 0.001 |
|  | 2 | Bangka Belitung Island | 21.6 | 4 | 77,138 | 56 | 16.2 | 128.9 | < 0.001 |
|  | 3 | Lampung | 17.0 | 7 | 158,466 | 55 | 15.9 | 61.3 | < 0.001 |
|  | 4 | West Sumatra | 108.7 | 10 | 64,950 | 31 | 9 | 78.0 | < 0.001 |
|  | 5 | North Sumatra | < 1 | 1 | 11,774 | 14 | 4 | 184.6 | < 0.001 |
|  | 6 | Riau Island | 30.3 | 5 | 37,966 | 12 | 3.5 | 48.8 | < 0.001 |
|  | 7 | North Sumatra | < 1 | 1 | 16,768 | 6 | 1.7 | 54.2 | < 0.001 |
|  | 8 | Lampung | < 1 | 1 | 26,377 | 5 | 1.4 | 28.6 | 0.009 |
|  | 9 | Aceh | < 1 | 1 | 4,477 | 3 | 0.9 | 100.7 | 0.022 |

Cluster number 1 indicates the primary cluster. Radius in kilometres (km). PHC = Primary health centre. RR = Relative risk.

Table S5 Summary of *P. vivax* clusters by year and province.

| **Year** | **Cluster** | **Province** | **Radius (km)** | **No of PHC** | **Pop** | **No of cases** | **% case** | **RR** | **p-value** |
| --- | --- | --- | --- | --- | --- | --- | --- | --- | --- |
| **2019** | 1 | Lampung | < 1 | 1 | 37,400 | 454 | 19.6 | 333.4 | < 0.001 |
|  | 2 | North Sumatra | < 1 | 1 | 28,921 | 228 | 9.8 | 193.1 | < 0.001 |
|  | 3 | North Sumatra | 9.1 | 4 | 142,092 | 248 | 10.7 | 43.1 | < 0.001 |
|  | 4 | Lampung | 7.6 | 14 | 443,995 | 224 | 9.7 | 12.2 | < 0.001 |
|  | 5 | North Sumatra | < 1 | 1 | 35,059 | 89 | 3.8 | 58.3 | < 0.001 |
|  | 6 | West Sumatra | < 1 | 1 | 37,586 | 84 | 3.6 | 51.2 | < 0.001 |
|  | 7 | Lampung | < 1 | 1 | 36,717 | 83 | 3.6 | 51.8 | < 0.001 |
|  | 8 | West Sumatra | < 1 | 1 | 7,454 | 47 | 2 | 142.2 | < 0.001 |
|  | 9 | South Sumatra | 21.8 | 8 | 215,843 | 114 | 4.9 | 12.2 | < 0.001 |
|  | 10 | Lampung | 14.2 | 3 | 66,106 | 73 | 3.2 | 25.2 | < 0.001 |
|  | 11 | North Sumatra | < 1 | 1 | 35,194 | 39 | 1.7 | 24.9 | < 0.001 |
|  | 12 | Jambi | < 1 | 1 | 16,845 | 31 | 1.3 | 41.2 | < 0.001 |
|  | 13 | Lampung | < 1 | 1 | 22,413 | 31 | 1.3 | 31.0 | < 0.001 |
|  | 14 | Bengkulu | < 1 | 1 | 16,144 | 28 | 1.2 | 38.8 | < 0.001 |
|  | 15 | Bengkulu | < 1 | 1 | 6,352 | 20 | 0.9 | 70.2 | < 0.001 |
|  | 16 | North Sumatra | 30.5 | 12 | 340,621 | 75 | 3.2 | 5.0 | < 0.001 |
|  | 17 | Lampung | < 1 | 1 | 52,685 | 24 | 1 | 10.2 | < 0.001 |
|  | 18 | Lampung | < 1 | 1 | 22,745 | 17 | 0.7 | 16.6 | < 0.001 |
|  | 19 | Bengkulu | < 1 | 1 | 12,747 | 13 | 0.6 | 22.7 | < 0.001 |
|  | 20 | Riau | < 1 | 1 | 22,550 | 15 | 0.6 | 14.8 | < 0.001 |
|  | 21 | North Sumatra | 9.3 | 6 | 128,065 | 30 | 1.3 | 5.2 | < 0.001 |
|  | 22 | Bengkulu | < 1 | 1 | 14,636 | 12 | 0.5 | 18.2 | < 0.001 |
|  | 23 | North Sumatra | < 1 | 1 | 20,470 | 9 | 0.4 | 9.7 | 0.01 |
|  | 24 | Bangka Belitung Island | 21.6 | 4 | 75,318 | 15 | 0.6 | 4.4 | 0.03 |
| **2020** | 1 | Riau | < 1 | 1 | 38,402 | 1693 | 53.8 | 1563.7 | < 0.001 |
|  | 2 | North Sumatra | 21.7 | 13 | 350,867 | 407 | 12.9 | 21.7 | < 0.001 |
|  | 3 | North Sumatra | 19.9 | 2 | 35,691 | 215 | 6.8 | 105.9 | < 0.001 |
|  | 4 | Riau Island | 24.8 | 3 | 20,547 | 147 | 4.7 | 122.9 | < 0.001 |
|  | 5 | Lampung | < 1 | 1 | 37,739 | 129 | 4.1 | 58.4 | < 0.001 |
|  | 6 | Bengkulu | 7.1 | 2 | 20,695 | 72 | 2.3 | 58.3 | < 0.001 |
|  | 7 | Aceh | < 1 | 1 | 9,324 | 51 | 1.6 | 91.1 | < 0.001 |
|  | 8 | North Sumatra | < 1 | 1 | 33,986 | 50 | 1.6 | 24.5 | < 0.001 |
|  | 9 | Bangka Belitung Island | 1.8 | 2 | 33,306 | 24 | 0.8 | 11.9 | < 0.001 |
|  | 10 | North Sumatra | < 1 | 1 | 16,768 | 17 | 0.5 | 16.7 | < 0.001 |
|  | 11 | Lampung | 14.2 | 3 | 66,367 | 28 | 0.9 | 7.0 | < 0.001 |
|  | 12 | Lampung | < 1 | 1 | 22,937 | 17 | 0.5 | 12.2 | < 0.001 |
|  | 13 | Lampung | < 1 | 1 | 37,180 | 17 | 0.5 | 7.5 | < 0.001 |
|  | 14 | Lampung | 2.1 | 3 | 68,367 | 19 | 0.6 | 4.6 | 0.002 |
|  | 15 | Jambi | < 1 | 1 | 17,420 | 10 | 0.3 | 9.4 | 0.003 |
|  | 16 | Bengkulu | < 1 | 1 | 6,352 | 6 | 0.2 | 15.5 | 0.038 |
| **2021** | 1 | North Sumatra | 48.9 | 18 | 418,029 | 1818 | 47.6 | 111.1 | < 0.001 |
|  | 2 | North Sumatra | 16.5 | 16 | 452,675 | 978 | 25.6 | 38.9 | < 0.001 |
|  | 3 | Lampung | 7.9 | 3 | 92,784 | 364 | 9.5 | 58.4 | < 0.001 |
|  | 4 | Aceh | < 1 | 1 | 9,324 | 170 | 4.5 | 257.5 | < 0.001 |
|  | 5 | North Sumatra | 7.8 | 4 | 73,885 | 90 | 2.4 | 16.8 | < 0.001 |
|  | 6 | Bangka Belitung Island | < 1 | 1 | 12,396 | 20 | 0.5 | 21.9 | < 0.001 |
|  | 7 | North Sumatra | < 1 | 1 | 16,768 | 16 | 0.4 | 12.9 | < 0.001 |
|  | 8 | Riau | 26.4 | 6 | 158,267 | 42 | 1.1 | 3.6 | < 0.001 |
|  | 9 | Riau Island | 24.8 | 3 | 21,728 | 16 | 0.4 | 10.0 | < 0.001 |
|  | 10 | Jambi | < 1 | 1 | 17,947 | 10 | 0.3 | 7.5 | 0.02 |

Cluster number 1 indicates the primary cluster. Radius in kilometres (km). PHC = Primary health centre. RR = Relative risk.

Table S6 Summary of probable *P. knowlesi* clusters by year and province.

| **Year** | **Cluster** | **Province** | **Radius (km)** | **No of PHC** | **Pop** | **No of cases** | **% case** | **RR** | **p-value** |
| --- | --- | --- | --- | --- | --- | --- | --- | --- | --- |
| **2019** | 1 | Aceh | < 1 | 1 | 12,262 | 25 | 25 | 1390.1 | < 0.001 |
|  | 2 | Aceh | 67.9 | 32 | 300,539 | 35 | 35 | 91.1 | < 0.001 |
|  | 3 | Lampung | < 1 | 1 | 36,154 | 9 | 9 | 139.8 | < 0.001 |
|  | 4 | Aceh | 8.3 | 4 | 25,512 | 8 | 8 | 174.3 | < 0.001 |
|  | 5 | Aceh | 76.4 | 44 | 442,728 | 16 | 16 | 21.8 | < 0.001 |
| **2020** | 1 | Aceh | 67.9 | 32 | 298,535 | 129 | 75 | 514.9 | < 0.001 |
|  | 2 | Aceh | < 1 | 1 | 13,391 | 18 | 10.5 | 449.7 | < 0.001 |
|  | 3 | Aceh | 63.9 | 36 | 411,918 | 11 | 6.4 | 8.5 | 0.002 |
|  | 4 | Aceh | < 1 | 1 | 18,514 | 4 | 2.3 | 66.3 | 0.004 |
|  | 5 | Aceh | 8.3 | 5 | 33,638 | 4 | 2.3 | 36.5 | 0.025 |
| **2021** | 1 | Aceh | 204.5 | 286 | 4,243,444 | 90 | 100 |  | < 0.001 |

Cluster number 1 indicates the primary cluster. Radius in kilometres (km). PHC = Primary health centre. RR = Relative risk.

Figure S1 Clusters of probable *P. knowlesi* in a) 2019, b) 2020, c) 2021. Red circles represent the primary cluster, and pink circles represent the secondary cluster. The point symbol represents the primary health care location, and the darker colour represents the higher incidence rate (per 1,000 population).

**References**

1. Badan Pusat Statistik Indonesia. Statistical Yearbook of Indonesia 2022. 2022. https://www.bps.go.id/en/publication/2022/02/25/0a2afea4fab72a5d052cb315/statistical-yearbook-of-indonesia-2022.html. Accessed 5 July 2023.

2. Badan Pusat Statistik Indonesia. Indeks Pembangunan Manusia 2021. 2022. https://www.bps.go.id/id/publication/2022/05/11/48b6466dcf14b562df9f17e2/indeks-pembangunan-manusia-2021.html. Accessed 1 July 2024.

3. Karra K, Kontgis C, Statman-Weil Z, Mazzariello JC, Mathis M, Brumby SP. Global land use / land cover with Sentinel 2 and deep learning. In: 2021 IEEE International Geoscience and Remote Sensing Symposium IGARSS. 2021. p. 4704–7. https://doi.org/10.1109/IGARSS47720.2021.9553499.
